# Upregulation of leukocyte immunoglobulin-like receptor B4 on interstitial macrophages in COPD; their possible protective role against emphysema formation

**DOI:** 10.1186/s12931-021-01828-3

**Published:** 2021-08-23

**Authors:** Ayumi Mitsune, Mitsuhiro Yamada, Naoya Fujino, Tadahisa Numakura, Tomohiro Ichikawa, Ayumi Suzuki, Shuichiro Matsumoto, Yoshiya Mitsuhashi, Koji Itakura, Tomonori Makiguchi, Akira Koarai, Tsutomu Tamada, Shota Endo, Toshiyuki Takai, Yoshinori Okada, Satoshi Suzuki, Masakazu Ichinose, Hisatoshi Sugiura

**Affiliations:** 1 Department of Respiratory Medicine, Tohoku University Graduate School of Medicine, 1-1 Seiryo-machi, Aoba-ku, Sendai, Miyagi 9808574 Japan; 2 Academic Center, Osaki Citizen Hospital, Osaki, Miyagi 9896183 Japan; 3 Department of Experimental Immunology, Institute of Development, Aging and Cancer, Tohoku University, Sendai, Miyagi 9808575 Japan; 4 Department of Thoracic Surgery, Institute of Development, Aging and Cancer, Tohoku University, Sendai, Miyagi 9808575 Japan; 5 Department of Thoracic Surgery, Japanese Red Cross Ishinomaki Hospital, Ishinomaki, Miyagi 9868522 Japan

**Keywords:** Pulmonary macrophage, Matrix metalloproteinase 12, Pulmonary emphysema

## Abstract

**Background:**

Leukocyte immunoglobulin-like receptor B4 (LILRB4) is one of the inhibitory receptors in various types of immune cells including macrophages. Previous reports suggested that LILRB4 could be involved in a negative feedback system to prevent excessive inflammatory responses. However, its role has been unclear in chronic obstructive pulmonary disease (COPD), in which macrophages play a crucial role in the pathogenesis. In this study, we aimed to examine the changes of LILRB4 on macrophages both in the lung specimens of COPD patients and the lungs of a mouse emphysema model. We then tried to compare the differences in both inflammation and emphysematous changes of the model between wild-type and LILRB4-deficient mice in order to elucidate the role of LILRB4 in the pathogenesis of COPD.

**Methods:**

We prepared single-cell suspensions of resected lung specimens of never-smokers (n = 21), non-COPD smokers (n = 16), and COPD patients (n = 14). The identification of LILRB4-expressing cells and the level of LILRB4 expression were evaluated by flow cytometry. We analyzed the relationships between the LILRB4 expression and clinical characteristics including respiratory function. In the experiments using an elastase-induced mouse model of emphysema, we also analyzed the LILRB4 expression on lung macrophages. We compared inflammatory cell accumulation and emphysematous changes induced by elastase instillation between wild-type and LILRB4-deficient mice.

**Results:**

The levels of surface expression of LILRB4 are relatively high on monocyte linage cells including macrophages in the human lungs. The percentage of LILRB4^+^ cells in lung interstitial macrophages was increased in COPD patients compared to non-COPD smokers (*p* = 0.018) and correlated with the severity of emphysematous lesions detected by CT scan (r_s_ = 0.559, *p* < 0.001), whereas the amount of smoking showed no correlation with LILRB4 expression. Increased LILRB4 on interstitial macrophages was also observed in elastase-treated mice (*p* = 0.008). LILRB4-deficient mice showed severer emphysematous lesions with increased MMP-12 expression in the model.

**Conclusions:**

LILRB4 on interstitial macrophages was upregulated both in human COPD lungs and in a mouse model of emphysema. This upregulated LILRB4 may have a protective effect against emphysema formation, possibly through decreasing MMP-12 expression in the lungs.

## Background

Chronic obstructive pulmonary disease (COPD) is a chronic respiratory disease caused by long-time inhalation exposure to toxic substances, mostly cigarette smoke [1]. The pathological features of COPD are characterized by both emphysematous changes of the lung parenchyma and inflammation of the small airways [2]. Pharmacological reagents, including bronchodilators and inhaled corticosteroids, are used to reduce symptoms, prevent exacerbations, and improve exercise tolerance and the health status of COPD patients [3]. Inhaled corticosteroids, combined with bronchodilators, are used for COPD patients as an anti-inflammatory therapy [3]. However, it is also true that chronic inflammation in COPD is steroid-resistant [4], so the effects of inhaled corticosteroids against persistent inflammation in COPD are limited. Therefore, further exploration for the mechanism of persistent inflammation and the inflammatory cells involved could be important to determine the new drug targets for anti-inflammatory therapy in COPD.

For further understanding of the pathogenesis of chronic inflammation in COPD, we focused on leukocyte immunoglobulin-like receptor B4 (LILRB4) in this study. LILRB4 is one of the inhibitory receptors expressed in various types of immune cells including monocytes and macrophages. LILRB4 is also called immunoglobulin-like transcripts 3 (ILT-3), CD85k, gp49B [5, 6]. Inhibitory receptors are characterized by an intracellular domain called immunoreceptor tyrosine-based inhibition motif (ITIM). Tyrosine phosphatase SHP-1 and SHP-2 are associated upon stimulation and suppress activation signals by the dephosphorylation of tyrosine-phosphorylated proteins [7, 8]. It has been reported that LILRB4 are involved in the pathogenesis of various diseases such as allergic diseases [9, 10], acute lung injury [11], cancer [12–15], autoimmune diseases [16], transplantation immunity [17, 18], nonalcoholic fatty liver disease [19], and infection disease [20]. It has been reported that LILRB4 on monocyte lineage cells including macrophages was upregulated by inflammatory stimuli including lipopolysaccharide [11, 21], suggesting that LILRB4 could be involved in a negative feedback system to prevent excessive inflammatory responses. However, little is known about the expression kinetics and role of LILRB4 in the pathogenesis of COPD.

In this study, we first aimed to analyze the expression of LILRB4 in lung single cells derived from surgically resected lungs of never-smokers, non-COPD smokers, and COPD patients. We examined which type of cells expressed LILRB4 and analyzed the relation of LILRB4 expression to the prevalence of COPD, respiratory function, smoking, and imaging findings. In addition, we analyzed the changes of LILRB4 expression in an elastase-induced mouse model of emphysema. We further examined the inflammatory responses and emphysematous changes in a mouse model of emphysema using both wild-type and LILRB4-deficietnt mice to surmise the role of LILRB4 in the pathogenesis of emphysematous lesions and COPD.

## Methods

### Study population

This study included 51 patients who received surgery for lung cancer in Ishinomaki Red Cross Hospital and Tohoku University Hospital. Patients with respiratory disease other than COPD were excluded. The diagnosis of COPD was determined based on the Global Initiative for Chronic Obstructive Lung Disease (GOLD) guidelines (https://goldcopd.org). The patients were divided into three groups: non-smokers, non-COPD smokers, and COPD patients. We used the Goddard score for the assessment of low attenuation areas (LAA) [22]. This study was approved by the Ethics Committee at Tohoku University School of Medicine (2017–1-352). Written informed consent was obtained from all patients.

### Preparation of human lung single-cell suspensions

We prepared human single-cell suspension as previously described with some modification [23–26]. Minced lung tissues were incubated with Hanks Balanced Salt Solution (Thermo Fisher Scientific, Waltham, MA, USA) containing 1.5 mg/ml Collagenase A (Sigma-Aldrich, St. Louis, MO) and 2000 KU/ml DNase I (Sigma-Aldrich) at 37 °C for 45 min, then minced again with scissors and incubated at 37 °C for 45 min. Single-cell suspensions were filtered with a 100 µm cell strainer (BD biosciences) twice and red blood cells were lysed with ammonium-chloride-potassium lysis buffer (Thermo Fisher Scientific). Cells were resuspended in RPMI 1640 medium (containing l-glutamine and 25 mM HEPES; Thermo Fisher Scientific) with 5% fetal bovine serum and 2% penicillin–streptomycin-amphotericin B suspension (100 units/ml penicillin, 100 µg/ml streptomycin, and 2.5 µg/ml, amphotericin B; FUJIFILM Wako Chemicals, Osaka, Japan) and filtered twice with 70 µm cell strainer (BD Biosciences, Franklin Lakes, NJ, USA). Cell numbers were calculated using trypan blue staining and analyzed by flow cytometry.

### Mice

C57BL/6 mice were purchased from Charles River Laboratories Japan (Yokohama, Japan) and 7 to 10-week-old female mice were used. LILRB4-deficient mice (gp49B^−/−^) with the B6 background were previously established [27]. All mice were maintained and bred in the Institute for Animal Experimentation, Tohoku University Graduate School of Medicine, under specific pathogen-free conditions. All animal protocols were reviewed and approved by the Animal Studies Committee of Tohoku University.

### Elastase-induced emphysema in mouse model

A mouse model of elastase-induced emphysema was prepared as previously described [26]. Briefly, mice were anesthetized with isoflurane temporarily and were given an intranasal instillation of 3 units porcine pancreatic elastase (FUJIFILM Wako Chemicals) in 50 µl of PBS or 50 µl of PBS alone. Analysis of macrophages was conducted on day 7 and histological examination and CT scan were conducted on day 21.

### Bronchoalveolar lavage (BAL)

Mice were injected intraperitoneally with triple mixed anesthesia of medetomidine hydrochloride (0.3 mg/kg), midazolam (4 mg/kg), and butorphanol tartrate (5 mg/kg). After euthanization by cutting the aorta, we inserted a 20-gauge needle into the trachea. Bronchoalveolar lavage fluid (BALF) was collected by washing the lungs three times with 1 ml of PBS. BALF was centrifuged at 300 rpm for 5 min and the supernatant was used for cytokine analysis. Cells were resuspended in PBS and cell counts were determined by a hemocytometer. Cell fractionation was calculated by cytospin slides stained by Diff-Quick method.

### Preparation of mouse lung single-cell suspensions

Mouse single-cell suspensions were prepared as previously described with some modification [28]. Lungs chopped with scissors were incubated at 37 °C for 45 min in RPMI solution containing 50 µg/ml Liberase TM (Roche, Basel, Switzerland) and 10 µg/ml DNase I (Roche). The lung tissue was passed through a 40 µm cell strainer. After centrifugation, the cell pellets were resuspended in ACK lysis buffer (Thermo-Fischer Scientific) and incubated to remove red blood cells. The samples were washed with PBS and resuspended in the staining buffer for flow cytometric analysis.

### Flow cytometry

Flow cytometry was performed as previously described with some modification [24, 29]. LIVE/DEAD Fixable Dead Cell Stain Kit (Invitrogen, Carlsbad, CA) was added to single-cell suspensions and incubated at 4 °C for 30 min. After resuspension in FACS buffer containing PBS with 0.1% sodium azide and 2% FBS, human FcR Blocking Reagent (Miltenyi Biotec) in the case of human and anti-CD16/32 mouse antibody in the case of mouse were added to prevent non-specific staining, then incubated 4 °C for 5 min. Based on previous reports [30–32], we defined human alveolar macrophages as FSC^high^CD45^+^CD206^+^CD14^−^ cells, human interstitial macrophages as FSC^mid^CD45^+^CD206^+^CD14^+^ cells, mouse alveolar macrophages as CD45^+^Ly6G^−^CD64^+^CD24^−^CD11b^int^CD11c^+^ cells and mouse interstitial macrophages as CD45^+^Ly6G^−^CD64^+^CD24^−^CD11b^+^CD11c^−^ cells. Data were collected by LSR Fortessa (BD Bioscience) and analyzed by FCS express 6 software (De Novo Software, Glendale, CA). Cell sorting was performed using FACS Aria II (BD Biosciences). We utilized fluorescence minus one controls to distinguish the positive population from the negative one.

### Antibodies

Brilliant Violet 421-conjugated anti-human CD45 (HI30), APC-conjugated anti-human CD3 (OKT3), APC-conjugated anti-human CD19 (HIB19), FITC-conjugated anti-human CD14 (M5E2), CD11c-conjugated anti-human CD11c (3.9), PE-Cy7-conjugated anti-human HLA-DR (LN3), PerCP-Cy5.5-conjugated anti-human CD56 (5.1H11), PE-conjugated anti-human LILRB4 (ZM4.1), Pacific blue-conjugated anti-mouse CD45 (30-F11), Brilliant violet 510-conjugated anti-mouse CD11b (M1/70), PerCP-Cy5.5-conjugated anti-mouse CD11c (N418), FITC-conjugated anti-mouse Ly6G (1A8), PE-Cy7-conjugated anti-mouse CD64 (X54-5/7.1), APC/Fire 750-conjugated anti-mouse CD24 (M1/69), APC-conjugated anti-mouse IA/IE (M5/114.15.2), PE-conjugated anti-mouse LILRB4 (H1.1), PE-conjugated American Hamster IgG control antibody (HKT888) were purchased from Biolegend. APC conjugated anti-human CD206 (19.2), APC-conjugated mouse IgG_1_κ control antibody (P3.6.2.8.1), PE-conjugated mouse IgG_1_κ control antibody (P3.6.2.8.1) were purchased from eBiosciences.

### Histological analysis

The lung was refluxed by injecting PBS from the right ventricle. The 10% neutral buffered formalin was injected from the trachea at a pressure of 30 cmH_2_O and the lung was fixed for 24 h. We entrusted the production of paraffin-embedded sections to Experimental Animal pathology Platform Section, Tohoku University. Emphysematous changes were evaluated by the mean liner intercept (MLI) [33].

### Image analysis

Under 2% isoflurane anesthesia, mouse chest CT scans were performed using an X-ray CT system for laboratory animals (LaTheta LCT-200; Hitachi Aloka Medical Ltd., Tokyo, Japan). Calibration was carried out according to the manufacturer’s protocol. The CT value of air was set to -1000 Housefield Units (HU), and water was set to 0 HU. Data were converted to DICOM files and analyzed by LaTheta software (version 3.22) and Image J software (National Institutes of Health, Frederick, MD). The quantitative evaluation of emphysema was performed using the percentage of low attenuation area, which was defined as the area from − 871 to − 610 HU [34].

### Quantitative polymerase chain reaction (qPCR)

RNA was extracted from sorting cells using RNeasy Micro Kit (Qiagen, Valencia, CA) and from lung tissue using RNeasy Mini Kit (Qiagen, Valencia, CA). cDNA was synthesized by the High Capacity RNA-to-cDNA Kit (Thermo Fisher Scientific). Quantitative PCR was conducted on StepOne Plus (Thermo Fisher Scientific) using SYBR Premix Ex Taq (TaKaRa, Kusatsu, Japan).

The levels of mRNA expression were evaluated by the comparative CT method and glyceraldehyde-3-phosphate dehydrogenase (GAPDH) was used as an endogenous control gene. The reference sample was one of the PBS-treated control samples or wild-type control samples. Primer sets were as follows: for mouse IL-10: forward, 5′-GCCAGAGCCACATGCTCCTA-3′, and reverse, 5′-GATAAGGCTTGGCAACCCAAGTAA-3′; for mouse IL-1β: forward, 5′-TCCAGGATGAGGACATGAGCAC-3′, and reverse, 5′-GAACGTCACACACCAGCAGGTTA-3′; for mouse Mmp12: forward, 5′-CTCTAGCCAGCACATGACTCCAA-3′, and reverse, 5′-CTGATGTGAAATGAGCCACACAAC-3′; for mouse TNF-α: forward, 5′-ACTCCAGGCGGTGCCTATGT-3′, and reverse, 5′-GTGAGGGTCTGGGCCATAGAA-3′; for mouse GAPDH: forward, 5′-TGTGTCCGTCGTGGATCTGA-3′, and reverse, 5′-TTGCTGTTGAAGTCGCAGGAG-3′.

### Statistical analysis

Data are expressed by a dot plot with median. Comparison between groups was performed using the Mann–Whitney U test. The Steel–Dwass test was used for nonparametric multiple comparisons among groups. To analysis the relationship between variables, Spearman’s rank correlation coefficient was calculated. All statistical analyses were conducted using GraphPad Prism version 7 (GraphPad Software Inc, Sac Diego, California, USA) or JMP Pro version 16 (SAS Institute Inc, Tokyo, Japan). *P* < 0.05 was considered significant.

## Results

### LILRB4 expression on interstitial macrophages was elevated in COPD patients

**Fig. 1:**
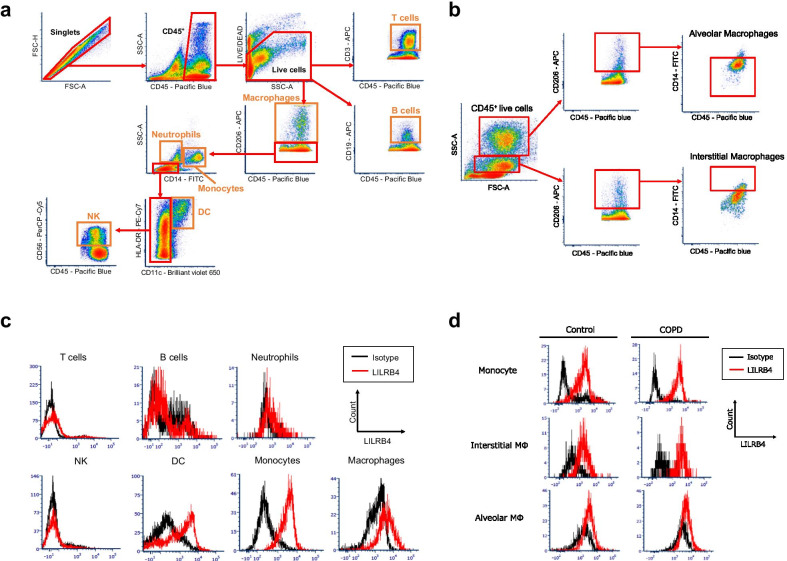
The expression of LILRB4 on lung interstitial macrophages was upregulated in COPD patients. **a** The flow cytometric gating strategy for detecting leukocyte lineages in human lung tissue. **b** The gating strategy of the flow cytometry for detecting human lung macrophages. **c** Representative histograms of LILRB4 expression on lung T cells, B cells, neutrophils, natural killer cells, dendritic cells, monocytes, and macrophages. Black line indicates staining with an isotype control antibody for anti-LILRB4. **d** Representative histograms of LILRB4 expression on monocyte, alveolar macrophages, and interstitial macrophages in the single cell suspension prepared from the lungs of never-smoker control or COPD patients. *NK* natural killer cells, *DC* dendritic cells, *COPD* chronic obstructive pulmonary disease

Because which types of leukocytes expressed LILRB4 in human lung were not clarified, we first examined the expression of LILRB4 on each type of leukocyte in single cell suspensions of normal lung tissue from the patients who received pneumonectomy for lung cancer. Among leukocytes, LILRB4 was clearly expressed on dendritic cells, monocytes, alveolar macrophages (AMs) and interstitial macrophages (IMs) (Fig. 1a–c). We then tried to examine whether the expression of LILRB4 was changed according to the smoking status and prevalence of COPD. We focused on monocytes and macrophages because it has been reported that these cells, especially lung macrophages, are involved in the pathogenesis of COPD [1]. We also had to exclude dendritic cells from further analyses because they were a relatively rare cell population and we could not harvest enough cells from every lung tissue sample for analyses. We compared LILRB4 expression on monocytes, AMs and IMs among non-smokers, non-COPD smokers, and COPD patients. The clinical characteristics of the human studies are shown in Table 1.

**Table 1:** Patients’ characteristics for analysis of LILRB4 expression on human lung macrophages

Characteristics	Never-smokers (n = 21)	Non-COPD smokers (n = 16)	COPD (n = 14)	*P* value
Age (years)	68.5 ± 8.7	67.6 ± 5.5	75.5 ± 3.7	< 0.001
Male/female	3/18	13/3	13/1	< 0.0001
Smoking (pack-years)	0	39.6 ± 29.2	48.4 ± 32.0	< 0.0001
FVC (L)	2.8 ± 0.7	3.2 ± 0.9	3.2 ± 0.5	0.017
FEV1 (L)	2.2 ± 0.6	2.6 ± 0.4	2.1 ± 0.3	0.004
FEV1/FVC (%)	83.9 ± 6.2	95.9 ± 11.0	63.5 ± 5.7	< 0.0001
FEV1 (% of predicted value)	108.5 ± 19.3	91.5 ± 8.6	82.6 ± 9.4	< 0.0001
% DLCO	97.1 ± 17.2	86.4 ± 19.5	93.1 ± 33.6	N.S
% DLCO/VA (% predicted value)	103.7 ± 16.3	93.5 ± 28.9	79.7 ± 23.4	N.S
GOLD stage (I/II)	–	–	11/3	

Data are presented as mean ± standard deviation or number. *FVC* forced vital capacity, *FEV*_*1*_ forced expiratory volume in 1 s, *DLCO* diffusing capacity of the lung for carbon monoxide, *GOLD* global initiative for chronic obstructive lung disease, *N.S.* not significant

**Fig. 2:**
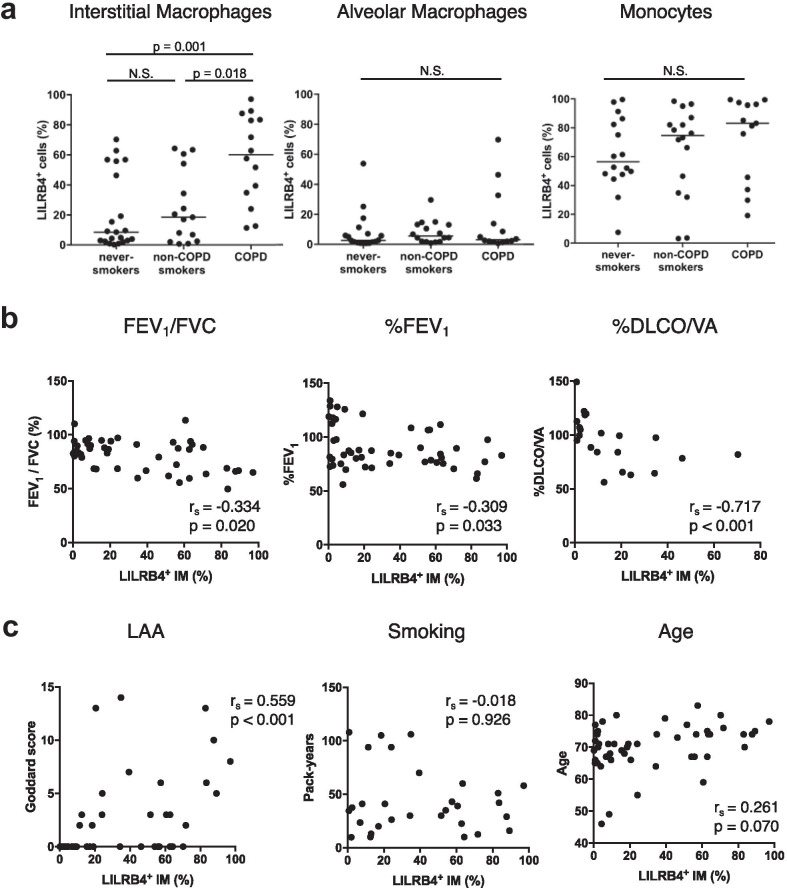
The correlation of the upregulation of LILRB4 expression on interstitial macrophages with the severity of emphysematous lesions. **a** Percentages of LILRB4 expression on interstitial macrophages (IMs, left), alveolar macrophages (AMs, middle), and monocytes (right) in the lungs from never-smokers, non-COPD smokers, and COPD patients. n = 21 for never-smokers, n = 16 for non-COPD smokers, n = 14 for COPD patients. Data are presented as median with a dot for each sample. The Steel–Dwass test was used for nonparametric multiple comparisons among the groups. N.S.: not significant. **b** Correlation analysis of the percentage of LILRB4 positive cells in lung IMs with FEV_1_/FVC (left), %FEV_1_ (middle), or %DLCO/VA (right). n = 51 for FEV_1_/FVC and %FEV_1_, n = 21 for %DLCO/VA. **c** Correlation analysis between the percentage of LILRB4 positive cells in lung IMs and Goddard score (left), smoking amount (middle), and age (right). N = 51 for each analysis. To analyze the relationship between variables, the spearman correlation coefficient was used. *FEV*_*1*_ forced expiratory volume in 1 s, *FVC* forced vital capacity, *DLCO* diffusing capacity of the lung for carbon monoxide, *VA* alveolar volume, *LAA* low attenuation area

The percentage of LILRB4-positive cells in IMs of the COPD patients was significantly higher than in those of non-smokers and non-COPD smokers (Figs. 1d and 2a). On the other hand, there were no significant differences in the percentages of LILRB4-positive cells in lung AMs and monocytes between the patient groups (Fig. 2a).

We next analyzed whether the percentage of LILRB4-positive cells in IMs was correlated with the patients’ characteristics including lung function and severity of emphysematous lesions. The percentage of LILRB4-positive cells in IMs was significantly associated with the FEV_1_/FVC ratio and percent predicted FEV_1_ (%FEV_1_) (Fig. 2b). The percent predicted DLCO/VA (%DLCO/VA) had a significant negative correlation with the percentage of LILRB4-positive cells in IMs (Fig. 2b). We then used the Goddard score to examine the severity of emphysematous lesions by analyzing low attenuation areas on CT images and their correlation with the existence of LILRB4-positive IMs. The severity of emphysematous lesions was well correlated with the percentage of LILRB4-positive cells in IMs (Fig. 2c). The percentage of LILRB4-positive cells in IMs did not significantly correlate with the smoking amount or age (Fig. 2c). These results suggested that the severity of emphysematous lesions was correlated with the accumulation of LILRB4-positive IMs.

### LILRB4-deficient mice show exacerbation of emphysema formation induced by elastase administration

**Fig. 3:**
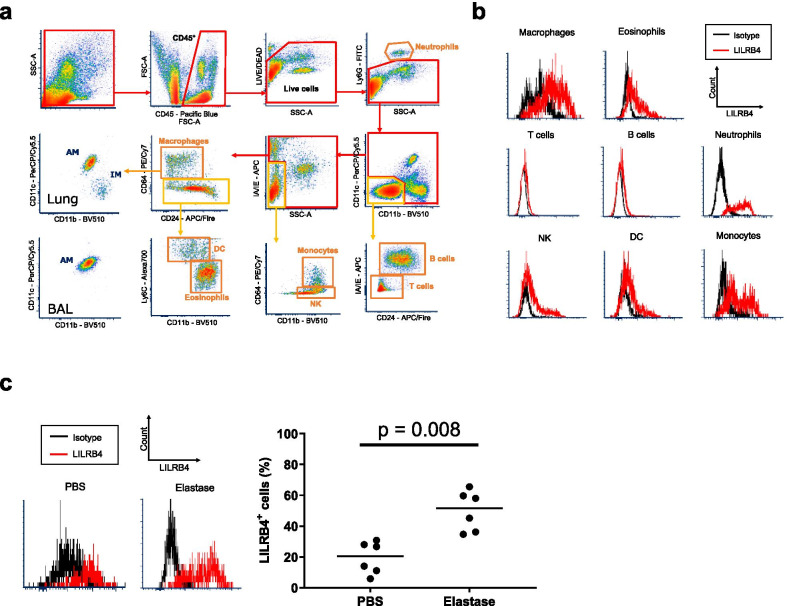
LILRB4 expression on interstitial macrophages in mouse lungs. **a** Flow cytometric gating strategy for detecting mouse lung hematopoietic cells. **b** Representative histograms of LILRB4 expression on macrophages, eosinophils, T cells, B cells, neutrophils, natural killer cells, dendritic cells, and monocytes. **c** Representative histograms of LILRB4 expression on IM (left) and the percentage of LILRB4 positive cells on IMs (right) from PBS-treated mice (n = 6) and elastase-treated wild-type mice (n = 6) seven days after the administration. Data are presented as median with a dot for each sample. Mann–Whitney U test was used for comparison between two groups. *AM* alveolar macrophages, *IM* interstitial macrophages, *NK* natural killer cells, *DC* dendritic cells

The correlation between the severity of emphysematous lesions and the accumulation of LILRB4-positive IMs in human lungs suggested that LILRB4 could have a role in the pathogenesis of emphysema. We then decided to elucidate whether LILRB4 was involved in the pathogenesis of emphysematous lesions. To answer this question, we utilized LILRB4-deficient (LILRB4^−/−^) mice and analyzed an elastase-induced emphysema mouse model. We first examined the LILRB4 expression on leukocytes in a mouse lung single cell suspension prepared by enzymatic digestion. In the mouse lungs, LILRB4 was also expressed on neutrophils, natural killer cells, and eosinophils, in addition to macrophages, monocytes and dendritic cells (Fig. 3a, b). We distinguished IMs from AMs by cell surface markers as previously reported [31] and examined the expression of LILRB4 on mouse IMs, finding that IMs expressing LILRB4 also existed in mouse lungs (Fig. 3a, c). We further examined the change of expression of LILRB4 on IMs in an elastase-induced emphysema mouse model. As in the lungs of human COPD patients, the percentage of LILRB4-positive cells in IMs significantly increased after the administration of elastase (Fig. 3c).

**Fig. 4:**
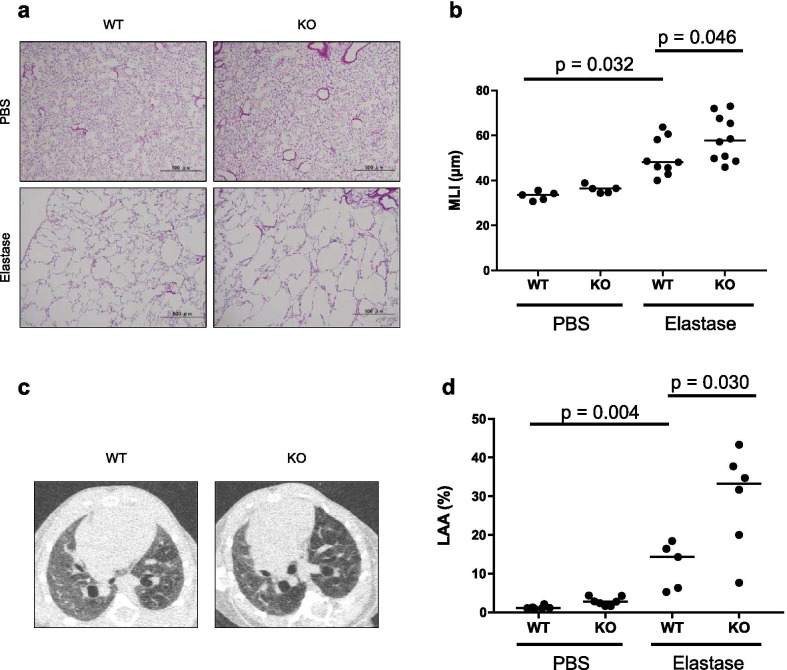
The deficiency of LILRB4 aggravated emphysematous lesions in the mouse elastase-induced emphysema model. **a** Representative hematoxylin/eosin-stained histological images of the lungs from wild-type or LILRB4^−/−^ mice on day 21 after PBS or elastase intranasal administration. Scale bar: 500 μm. **b** Mean liner intercepts calculated by analyzing histological images on day 21 after administration. **c** Representative chest CT images of wild-type mice (left) and LILRB4^−/−^ mice (right) on day 21 after intranasal administration of elastase. **d** Low attenuation area (LAA) of CT images on day 21 after administration. Data are presented as median with a dot for each sample. The Steel–Dwass test was used for nonparametric multiple comparisons among the groups. N = 5 to 10 per groups. *WT* wild-type mice, *KO* LILRB4-deficient mice, *MLI* mean liner intercept, *LAA* low attenuation area

We then examined the emphysema formation and the accumulation of inflammatory cells in the airway of the elastase-induced emphysema model. We measured both the mean linear intercept (MLI) by histopathological analysis and the percentage of the low attenuation area (LAA) in the lung field by CT at 21 days after the elastase administration. We found that both indicators, MLI and LAA, were significantly higher in LILRB4^−/−^ mice than to those in wild-type mice, indicating that the emphysematous lesion was exacerbated by the deficiency of LILRB4 (Fig. 4a–d). These data suggested that LILRB4 may have a protective function against emphysema formation in the elastase-induced mouse model.

**Fig. 5:**
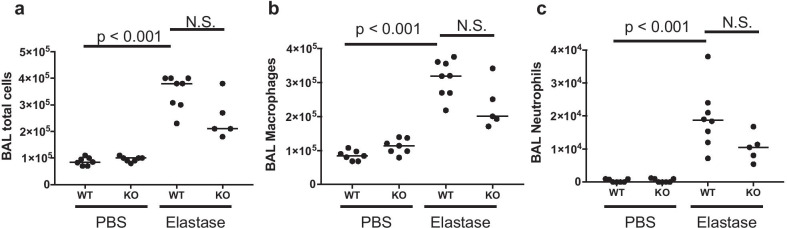
The deficiency of LILRB4 did not affect the accumulation induced by intranasal administration of elastase. BAL was performed on day 7 after the administration of PBS or elastase. The number of total cells (**a**), macrophages (**b**), and neutrophils (**c**) in BAL fluids were examined. Data are presented as median with a dot for each sample. The Steel–Dwass test was used for nonparametric multiple comparisons among the groups. N = 5 to 8 per groups. *WT* wild-type mice, *KO* LILRB4-deficient mice

**Fig. 6:**
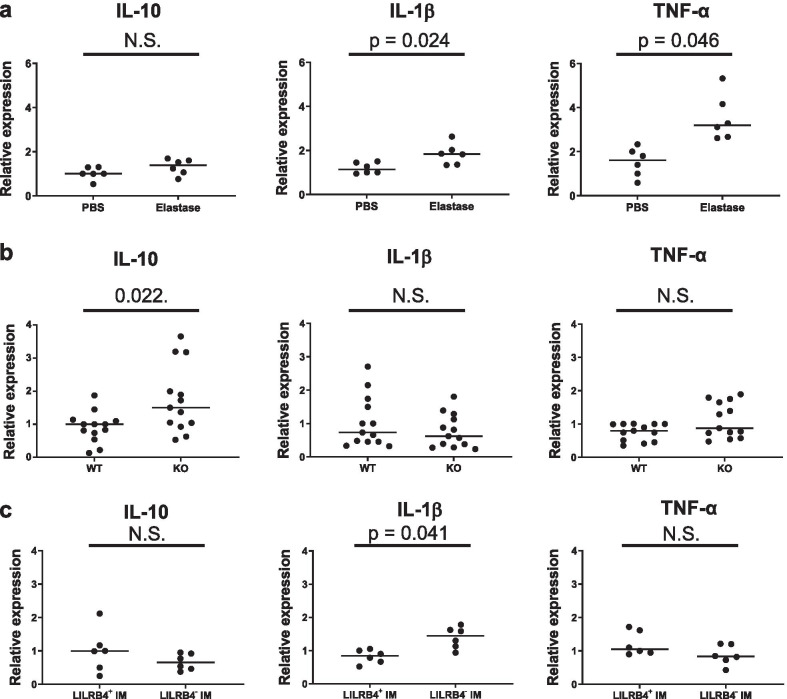
LILRB4-deficient mice enhanced IL-10 mRNA expression. **a** We administered PBS or elastase to wild-type mice and compared IL-10 (left), IL-1β (middle) and TNF-α (right) mRNA expressions. N = 6 per groups. The reference sample was one of the PBS-treated control samples. **b** Comparison of IL-10 (left), IL-1β (middle) and TNF-α (right) mRNA expressions between wild-type and LILRB4^−/−^ mice on day 7 after elastase administration. N = 13 per groups. The reference sample was one of the wild-type samples. **c** Comparison of IL-10 (left), IL-1β (middle) and TNF-α (right) mRNA expressions between LILRB4-positive IMs and LILRB4-negative IMs on day 7 after elastase administration. N = 6 per groups. The reference sample was one of the wild-type samples. Data are presented as median with a dot for each sample and analyzed by Mann–Whitney U test. *WT* wild-type mice, *KO* LILRB4-deficient mice, *IM* interstitial macrophages

We also examined inflammatory cell accumulation in the airway because it was possible that the exacerbated emphysema in LILRB4^−/−^ mice might have been due to the exacerbated inflammation induced by elastase. We performed BAL on day 7 and examined the number of the cells in BAL fluids. The numbers of total cells, as well as macrophages and neutrophils, were significantly elevated in the BAL fluid by elastase administration in both the wild-type and LILRB4^−/−^ mice. However, there were no significant differences in the cell counts between the wild-type and LILRB4^−/−^ mice (Fig. 5a–c). We further examined the levels of mRNA expression from pro-inflammatory cytokines (IL-1β and TNF-α) and an anti-inflammatory cytokine (IL-10) in the whole lungs of an emphysema model. The levels of IL-1β and TNF-α, but not those of IL-10, were significantly increased by the administration of elastase (Fig. 6a). In an elastase-induced emphysema model, the level of IL-10, but not those of inflammatory cytokines, was higher in LILRB4^−/−^ mice compared with wild-type mice (Fig. 6b). We also examined isolated lung IMs to determine the levels of cytokine mRNA because it has been reported that lung IM is a major producer of IL-10 [35]. Isolated IMs from elastase administered LILRB4^−/−^ mice showed similar levels of IL-10 and TNF-α, although they showed a higher level of IL-1β (Fig. 6c). These data suggested that the exacerbation of emphysema in LILRB4^−/−^ mice was not due to the aggravation of inflammatory cell accumulation and inflammatory cytokine production.

### The deficiency of LILRB4 enhanced MMP-12 production in a mouse emphysema model

**Fig. 7:**
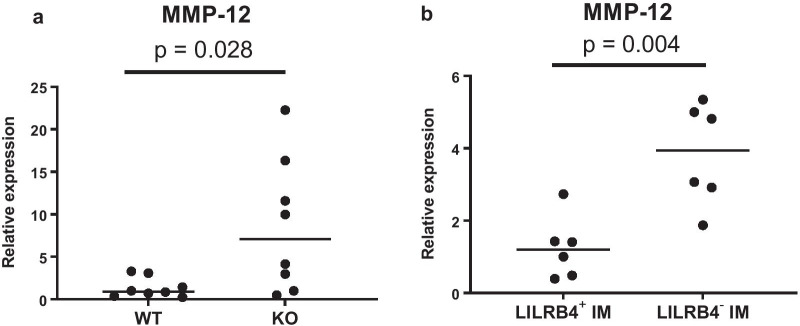
LILRB4-decifient mice enhanced MMP-12 mRNA expression. **a** MMP-12 mRNA expression in the whole lung between wild-type mice and LILRB4^−/−^ mice on day 7 after elastase administration. The mean value of WT was set to 1. N = 8 per groups. **b** MMP-12 mRNA expression between LILRB4-positive IMs and LILRB4-negative IMs. N = 6 per group. Data are presented as median with a dot for each sample. Statistical comparisons were analyzed by Mann–Whitney U test. *WT* wild-type mice, *KO* LILRB4-deficient mice

To explore the cause of the exacerbated emphysematous lesions in LILRB4^−/−^ mice, we focused on matrix metalloprotease 12 (MMP-12). MMP-12 is a protease associated with emphysema that is elevated in the sputum and BAL of COPD patients [36–39]. In animal models, emphysema is not induced by smoking stimulation in MMP-12 knockout mice [40]. A recent investigation has revealed that IM rather than AM is the major producer of MMP-12 in lungs [41]. We examined the expression level of MMP-12 mRNA in whole lungs. The expression level of MMP-12 mRNA in the whole lung of LILRB4^−/−^ mice in the emphysema model was significantly higher than that in wild-type mice (Fig. 7a). We then isolated IMs from digested lungs of the emphysema model and examined the levels of MMP-12 mRNA. LILRB4^−/−^ IMs had significantly higher levels of MMP-12 mRNA than wild-type IMs (Fig. 7b). These results suggested that the deficiency of LILRB4 enhanced the upregulation of MMP-12 by IMs in the lungs of the emphysema model, which may have caused the exacerbation of emphysematous lesions.

## Discussion

In this study, we focused on LILRB4, one of the inhibitory immune-receptors, and tried to determine the changes in expression of LILRB4 and its roles in the pathogenesis of human COPD and the development of emphysema in mice. Our analyses of both the human lung samples and LILRB4-deficeint mice suggest that LILRB4, which is upregulated on lung IMs in COPD patients, may have a protective effect against emphysema formation through decreasing MMP-12 expression.

It has been well reported that lung macrophages are involved in the pathogenesis of emphysematous lesions in both human and animal models [42]. Recent studies have revealed that there are IMs in addition to AMs, the classical lung macrophages, in the lungs of mouse and human, and IMs have distinctively different functions than AMs [43, 44]. It has been recently reported that IM is a major producer of MMP-12 in the lungs in a mouse emphysema model [41]. Our results suggest that LILRB4, which is upregulated on lung IMs in both human COPD patients and a mouse elastase-induced emphysema model, has a protective effect against the formation of emphysematous lesions through the attenuation of MMP-12 production, mainly by IMs.

We have not elucidated how the intracellular signaling via LILRB4 attenuates the expression of MMP-12 in lung IMs. The previous study has elucidated that IL-4 produced by basophils induces the differentiation of accumulated monocytes into MMP-12 producing IMs in a mouse model of emphysema induced by elastase [41]. The observations using genetically engineered mice, including the mice deficient for IL-4 specifically in basophils, have revealed that basophils play a critical role in emphysema formation via producing IL-4, which promotes the differentiation of the infiltrating monocytes into MMP-12 producing IMs in the lungs [41]. STAT6-deficient mice did not show the upregulation of MMP12 mRNA induced by IL-4 in bone marrow-derived macrophages, and chromatin immunoprecipitation-qPCR analyses revealed that IL-4 induced STAT6 binding to the promoter region of MMP12, suggesting that IL-4 mediates MMP-12 expression through STAT6 activation [45]. A protein-tyrosine phosphatase SHP-1 is recruited to LILRB4 through its ITIMs upon crosslinking [5]. A previous study reported that overexpression of SHP-1 reduced both the IL-4-dependent STAT6 activation and STAT6-mediated upregulation of IL-4 responsive genes [46]. Therefore, it is possible that LILRB4 attenuates the expression of MMP-12 in lung IMs through SHP-1 activation recruited to its ITIMs by the inhibition of STAT6 activation by IL-4 that is produced by basophils [41] and contribute to a protective effect against the formation of emphysematous lesions.

Our studies found that the percentage of LILRB4-positive cells in total lung IMs was significantly increased in both COPD patients and a mouse model of emphysema. We have not yet obtained clear evidence to explain how LILRB4 is upregulated on lung IMs during COPD. However, both previous reports [11, 19, 21] and an analysis of the promoter and enhancer region of LILRB4 provided in the public database likely suggests that LILRB4 may be upregulated by inflammatory stimuli including cytokines. A previous report showed that LILRB4 is upregulated on hepatocytes in nonalcoholic fatty liver disease (NAFLD), a chronic inflammatory disease of the liver [19]. In a high-fat diet induced NAFLD model in mice, the hepatocytes on which LILRB4 is upregulated by inflammatory stimuli showed an improvement from insulin resistance, glucose metabolic disorder, hepatic lipid accumulation, as well as inflammatory responses [19]. This negative feedback-loop is operated by SHP-1 recruitment to LILRB4 to inhibit TRAF6 ubiquitination and subsequent inactivation of NF-κB and mitogen‐activated protein kinase cascades, which results in the attenuation of inflammatory responses [19]. The fact that there are binding sites for both AP-1 and NF-κB, which are the transcription factors activated by inflammatory cytokines (https://www.genecards.org/), also supports the idea that LILRB4 is upregulated by inflammatory stimuli and operates the negative feedback through SHP-1.

This study has several limitations. First, for a human study, the sample size is relatively small and the COPD group had only GOLD stage I and II patients. However, analyses of single lung cells harvested from human lung samples including the lungs of COPD patients are valuable for exploring the pathogenesis of actual human COPD and emphysematous lesions, even if the sample size is small. Second, a mouse model of emphysema induced by elastase, in which both inflammation and the subsequent formation of emphysematous lesions were subacute, is not an optimal model for human COPD as compared to a cigarette smoke-induced model, although this animal model also shows the upregulation of MMP-12, as found in human COPD patients. Third, although fibronectin has been quite recently identified as a physiological ligand on both human monocytic leukemia cell line THP-1 cells and human primary monocytes [47], the existence of a pathophysiological ligand for LILRB4 on lung IMs in COPD and a mouse emphysema model remains unclear. Further examinations are needed to determine the ligand in order to understand the roles of LILRB4 and its ligand in the pathogenesis of emphysema.

In summary, the severity of emphysematous lesions was correlated with the accumulation of LILRB4-positive IMs in COPD patients. The deficiency of LILRB4 exacerbated emphysematous lesions in a mouse model of emphysema. The deficiency of LILRB4 enhanced the production of MMP-12 by lung IMs, which may contribute to the aggravation of emphysematous lesions. Therefore, LILRB4 may have a protective effect against emphysema formation by its involvement in a negative-feedback loop. Further investigation for LILRB4 and its ligand on IMs may elucidate the pathophysiology of COPD and its emphysematous lesions and possibly lead to the discovery of therapeutic targets for COPD.

## Data Availability

The datasets used and analysed during the current study are available from the corresponding author on reasonable request.

## Abbreviations

AM: Alveolar macrophage
BAL: Bronchoalveolar lavage
BALF: Bronchoalveolar lavage fluid
COPD: Chronic obstructive pulmonary disease
CT: Computed tomography
DLCO: Diffusing capacity of the lungs for carbon monoxide
FEV_1_: Forced expiratory volume in one second
%FEV_1_: Percent predicted forced expiratory volume in one second
FVC: Forced vital capacity
GOLD: Global initiative for chronic obstructive lung disease
IL-1: Interleukin-1β
IL-10: Interleukin-10
ILT-3: Immunoglobulin-like transcripts 3
IM: Interstitial macrophage
LAA: Low attenuation area
LILRB4: Leukocyte immunoglobulin-like receptor B4
MLI: Mean linear intercept
MMP-12: Matrix metalloprotease 12
NAFLD: Nonalcoholic fatty liver disease
STAT6: Signal transducer and activator of transcription 6
SHP-1: SH2-containing protein tyrosine phosphatase-1
TNF-α: Tumor necrosis factor-α
TRAF6: TNF receptor-associated factor 6
VA: Alveolar volume
